# Impact of social capital, harassment of women and girls, and water and sanitation access on premature birth and low infant birth weight in India

**DOI:** 10.1371/journal.pone.0205345

**Published:** 2018-10-08

**Authors:** Kelly K. Baker, William T. Story, Evan Walser-Kuntz, M. Bridget Zimmerman

**Affiliations:** 1 Department of Occupational and Environmental Health, University of Iowa College of Public Health, IA, United States of America; 2 Department of Community and Behavioral Health, University of Iowa College of Public Health, IA, United States of America; 3 Department of Biostatistics, University of Iowa College of Public Health, IA, United States of America; University of Michigan Medical School, UNITED STATES

## Abstract

**Background:**

Globally, preterm birth (PTB) and low infant birth weight (LBW) are leading causes of maternal and child morbidity and mortality. Inadequate water and sanitation access (WASH) are risk factors for PTB and LBW in low-income countries. Physical stress from carrying water and psychosocial stress from addressing sanitation needs in the open may be mechanisms underlying these associations. If so, then living in a community with strong social capital should be able to buffer the adverse effects of WASH on birth outcomes. The objective of this study is to assess the relationships between WASH access and social conditions (including harassment and social capital) on PTB and LBW outcomes among Indian women, and to test whether social conditions modified the association between WASH and birth outcomes.

**Methods and findings:**

This cohort study examined the effect of pre-birth WASH and social conditions on self-reported PTB status and LBW status for 7,926 women who gave birth between 2004/2005 and 2011/2012 Waves of the India Human Development Survey. PTB and LBW occurred in 14.9% and 15.5% of women, respectively. After adjusting for maternal biological and socioeconomic conditions, PTB was associated with sharing a building/compound latrine (Odds Ratio (OR) = 1.55; 95% Confidence Interval (CI) = 1.01, 2.38) versus private latrine access, but suggested an effect in the opposite direction for sharing a community/public latrine (OR = 0.67; CI = 0.45, 1.01). Open defecation, type of drinking water source, minutes per day spent fetching water, and one-way time to a drinking water source were not associated with PTB. LBW was associated with spending more than two hours per day fetching water compared to less than two hours (OR = 1.33; CI = 1.05, 1.70) and suggested an association with open defecation (OR = 1.22; CI = 1.00, 1.48), but was not associated with other types of sanitation, type of drinking water source, or time to a drinking water source. Harassment of women and girls in the community was associated with both PTB (OR = 1.33; CI = 1.09, 1.62) and LBW (OR = 1.26; CI = 1.03, 1.54). The data also showed a possible association of local crime with LBW (OR = 1.30; CI = 1.00, 1.68). Statistically significant (p<0.05) evidence of effect modification was only found for collective efficacy on the association between type of sanitation access and PTB. In addition, stratified analyses identified differences in effect size for walking time to the primary drinking water source and PTB by crime, sanitation access and PTB by harassment, and total hours per day fetching water and LBW by collective efficacy. Limitations of this observational study include risk of bias, inability to confirm causality, reliance on self-reported outcomes, and limited sub-group sample sizes for testing effect modification.

**Conclusions:**

The relationship between adverse birth outcomes and sanitation access, domestic water fetching, crime, and gender-based harassment suggests physical and psychosocial stress are possible mechanisms by which WASH access affects PTB and LBW among Indian women. Interventions that reduce domestic responsibilities related to water and sanitation and change social norms related to gender-based harassment may reduce rates of PTB and LBW in India.

## Introduction

Increasing the number of women who successfully carry a healthy, nourished infant to term is critical for achieving the Sustainable Development Goals (SDGs). Spontaneous preterm birth (PTB), caused by onset of labor or rupture of the fetal membranes before 37 completed weeks' gestation, is one of the leading global causes of death in neonates and children under five years of age, as well as maternal complications (e.g. placental previa and mortality) [1]. An estimated 12.9 to 15 million PTBs occur each year, with 60 to 85% of these births occurring in Asia and sub-Saharan Africa [2, 3]. Low birth weight (LBW), whether term or preterm status, is an even more common public health issue. In 2012, an estimated 23.3 million infants were born small for gestational age in low- and middle-income countries (LMICs) alone, of which 12.2 million infants were LBW (< 2,500 grams) [4]. Infants in LMICs who are born prematurely or at LBW experience a high risk of death, and are more likely to experience gastrointestinal infections, respiratory infections, growth stunting, and cognitive impairment in later childhood [5–12]. Critically, PTB rates appear to be rising [2], suggesting morbidity and mortality caused by PTB and LBW may rise as well. Early implementation of preventive interventions with women who are at high risk for PTB or LBW in the pregnancy period, before delivery occurs, could reduce a substantial amount of morbidity and mortality among newborns in LMICs [13].

A broad range of risk factors have been linked to PTB and LBW outcomes, including but not limited to maternal reproductive history, race, age, low antenatal care attendance, maternal malnutrition, vaginal and urinary tract infections, history of still birth, and anemia [14–16]. In LMICs, the struggle to address basic water, sanitation, and hygiene (WASH) needs may also be a major contributor to adverse birth outcomes. Two studies have linked unsafe WASH conditions with adverse birth outcomes. In Nigeria, women living in a house that shared a sanitation facility were more likely to experience PTB or LBW compared to women not sharing a facility, after adjusting for maternal obstetric conditions and maternal poverty indicators [17]. In India, women who defecated in the open or used available latrines infrequently, or who bathed in surface water sources were more likely to experience PTB and LBW [18]. These support a role for WASH in the global burden of PTB and LBW disease, although the mechanisms remain unclear.

The mechanisms through which WASH affects birth outcomes are potentially multifactorial. Inadequate WASH access can increase the risk of diarrheal and helminthic infections, as well as maternal malnutrition and mortality [19]. Yet, recent studies have expanded the scope of WASH-related diseases to include other PTB and LBW risk factors: reproductive tract infection symptoms, musculoskeletal injury, and maternal psychosocial stress [20–24]. Maternal psychosocial stress, depression, and anxiety, caused by both domestic and neighborhood-level social and environmental deprivation, are considered leading causes of PTB in high-income countries (HICs) [16, 25–29]. Evidence is lacking on the role of stress–especially community rather than familial sources of stress–in birth outcomes of LMIC women. The causes of stress are likely different than for women in HICs. Pregnant women in LMIC settings who live in chronic poverty must regularly engage in physically demanding domestic labor related to their gender, such as water fetching [24, 30]. The physical stress this places upon their bodies can be amplified if they must travel over longer, rougher terrain to acquire water or to find a place to defecate [24]. If water sources or defecation locations lie outside the home, women may also have to navigate socially stressful environments where they experience crime or harassment, in addition to the physical demands of labor [21, 22]. These physically and socially stressful living conditions could cause women’s bodies to increase expression of corticotrophin releasing hormone and immune inflammatory markers, which trigger secretion of prostaglandins and interleukins that stimulate myometrium contractions and/or rupture of chorioamniotic membranes resulting in premature labor [31].

Determining whether WASH-related stress affects birth outcomes for women in LMICs is critical for understanding whether the global prevalence of PTB and LBW could be reduced by improving WASH conditions for pregnant women. Studies in LMICs on maternal depression, anxiety, and stress and birth outcomes have focused mostly on intimate partner violence, stress, or abuse [32–35]. Only one study has linked premature labor to psychological stress caused by social conflict and violence, but in an extreme political conflict [36]. A universal index of PTB-inducing stress for LMIC women might be the daily physical demands of gender-based domestic labor when resources lie beyond the home and the social conflict and violence that occurs because of inadequate WASH resources within a neighborhood or village.

If stressful WASH conditions increase the risk of adverse birth outcomes, then social capital that reduces a woman’s domestic responsibilities, or exposure to social stressors while fetching water or practicing open defecation, may buffer the effect of inadequate WASH conditions on birth outcomes [37]. Past studies have shown that collective efficacy and social cohesion within communities are negatively associated with violence, crime, and harassment [38, 39]. In the social capital literature, collective efficacy and social cohesion are often referred to as the “social cohesion” perspective [40] or cognitive social capital [41]. This form of social capital is viewed as a collective attribute and is often subjectively verified by assessing attitudes and perceptions about one’s community. Social capital can affect sanitation behaviors and water access in several ways. First, social capital can enforce norms related to sanitation behaviors, such as using a latrine to defecate instead of defecating in open spaces [42]. Second, if women prefer to defecate in the open, social capital can reduce stress related to safety and privacy during open defecation by going out in groups [43]. Third, communities with high levels of trust and collective efficacy are better able to mobilize a joint response to local water issues [44], whereas divisive communities are not able to cooperate and mobilize resources to address water access [45]. Fourth, social capital can help women who must walk long distances and are at risk of assault and sexual abuse by creating a sense of solidarity and promoting collective action to walk together [30, 37]. Therefore, WASH interventions that build upon collective efficacy and social cohesion to reduce physical and psychosocial stress during pregnancy could reduce the global PTB and LBW disease burden.

In spite of the overwhelming evidence that stress leads to PTB in high-income populations, and evidence that poor WASH access causes stress for pregnant women, there are no studies explicitly examining the effect of WASH-related stress on birth outcomes in LMIC women. The objective of this study is to (1) investigate the relationship between WASH access and social conditions before birth and PTB and LBW outcomes, and (2) test whether social conditions modify the effect of WASH access on birth outcomes.

## Materials and methods

### Study population

India has one of the highest burdens of neonatal mortality caused by PTB and LBW [4]. The India Human Development Survey (IHDS) is a nationally representative survey of 33,510 women aged 15–49 in 2,474 villages or urban neighborhoods across 33 states and union territories of India [46]. This cohort study used data from women who gave birth to a child between the 2004/2005 and 2011/2012 waves of the survey. Household and individual (of women) survey datasets were linked by subject identification numbers across both waves of the survey. Inclusion criteria were being a woman who participated in both survey waves, who reported giving birth to a child between 2004/2005 and 2011/2012, and who had no missing data for PTB or LBW of the most recently born child.

### Outcome variables

The outcome variables for this study were extracted from the 2011/2012 survey on the birth status of the most recent child born after 2005 (if multiple children born between survey waves). Variables included (1) a binary variable for whether the participant reported premature labor during the birth of the child, and (2) a binary variable representing maternal perception that the birth weight of the child was small or very small, relative to a normal or above average infant (S1 Table).

### Environmental exposure variables

Information on WASH conditions was extracted from the 2004/2005 surveys to represent conditions prior to (preconception period) or during pregnancy. Four WASH variables were selected based upon their potential to be physically or psychosocially stressful during pregnancy (S1 Table). Type of drinking water source was a binary variable, for surface water (i.e., river, canal, pond, or stream) and water from a truck versus piped, hand pump, tube well, covered well, and rainwater sources. Time spent traveling to fetch drinking water from the primary household source was measured as (1) water source in household (reference), (2) between one and 15 minutes one way, and (3) more than 15 minutes one-way. The daily physical burden for fetching water for all domestic purposes was measured by the amount of time in minutes spent per day by women collecting and fetching water. The responses for women who fetched water from sources outside the household was normally distributed with a median of two hours, so this variable was collapsed into a binary variable as less than two hours per day versus greater than or equal to two hours per day. Sanitation access was defined using a modification of the World Health Organization/UNICEF Joint Monitoring Program criteria, which included (1) private household access to an improved sanitation facility (reference), (2) use of shared facility within the building or compound, (3) shared facility outside the building or compound *or* public latrine (combined due to small sample sizes), and (4) open defecation.

### Social exposure variables

Information on social conditions was extracted from the 2004/2005 surveys to represent conditions prior to (preconception period) or during pregnancy. Two questions measured the general safety of the woman’s village or neighborhood (S1 Table). Gender-specific harassment was measured with “How often are unmarried girls harassed in your village/urban neighborhood?” This item was measured using a three point scale for “never/rarely”, “sometimes”, or “often”, but was collapsed into a binary variable for “sometimes” or “often” versus “never”. Local crime was measured as a binary variable where a local crime was considered present if a positive response was provided to any one of three following questions:(1) “During the last twelve months, did anyone attack or threaten you or someone in your household?”; (2) During the last twelve months, was anything stolen that belonged to you or to somebody in your household?”; or (3) “During the last twelve months, did anyone break into your home or illegally get into your home?”. These two questions about safety used safer communities as the reference group.

Two questions assessed aspects of cognitive social capital within each village or neighborhood. Collective efficacy was measured using a binary variable based on the following question: “In some communities, when there is a water supply problem, people bond together to solve the problem. In other communities, people take care of their own families individually. What is your community like?” Respondents had two response options: bond together to solve problem versus each family solves problems individually. Social cohesion was measured using a binary variable based on the following question: “In this village/neighborhood, do people generally get along with each other or is there some conflict or a lot of conflict?”, contrasting “some conflict” and “a lot of conflict” against “none”. The social capital variables used greater levels of social capital as the reference group.

### Confounders

Factors that have previously been shown to be related to birth outcomes, and that may be confounders of the relationship between exposures and outcomes included: geography, household wealth index rank, female education index, religion, mother’s age, age at menarche, parity, history of stillbirth, maternal utilization of antenatal checkups, and maternal use of iron supplements during pregnancy (S1 Table). We intended to include caste in the analysis, but a substantial number of responses were missing. Out of concern that many of the missing responses might be persons of lower castes who felt shame or embarrassment at providing this information, we decided to not adjust for caste in the analysis, rather than potentially skew study effects by including only persons with caste data. Information on household-level socio-demographic factors was extracted from the 2004/2005 surveys to represent conditions prior to or at the time of pregnancy, while information related to parity, history of stillbirths, antenatal checkups, and iron supplementation was extracted from the 2011/2012 surveys to represent the respondent’s care during her most recent pregnancy. Geography was assessed as rural, urban, and urban slum. The household wealth index variable was created by factor analysis of 30 household assets to create a household asset score, which was then divided into the poorest, lower-middle income, upper-middle income, and wealthiest quartiles. Female education index was divided into no education, standards 1–9, and standard 10-college graduate. Religion was divided into Hindu versus other. Mother’s age, age at menarche, and parity were continuous variables. Recent history of stillbirth was a binary variable for a woman experiencing a stillbirth between the 2004/2005 survey and the 2011/2012 survey versus no stillbirth. Antenatal care was a binary variable for a woman reporting attending at least one antenatal care checkup versus no antenatal care. Iron supplementation was divided into no use, supplementation for less than three months, and supplementation for greater than three months. Although venting in the household cooking area is an important risk factor for PTB, we did not adjust for this confounder due to exceedingly large amounts of missing data that resulted in a 15% reduction in our final sample size (from 7,926 to 6,716 eligible subjects) when combined with other variables with missing data. In a separate analysis (data not shown), we adjusted for venting of the cooking area and did not find any substantive differences.

### Statistical analysis

Descriptive statistics are reported as percent, medians, and counts. Strength of association between WASH and social exposures on occurrence of self-reported PTB and LBW are estimated using outcome-specific generalized linear mixed model (SAS Version 9.4, proc glimmix) for a binary outcome with logit link function, and a random intercept term to account for variance between states. Effects are reported as odds ratios (OR) and 95% confidence intervals (95% CI). To assess the amount of variance in PTB and LBW outcomes that was accounted for by variation between states, the intra-class correlation coefficients (ICC) of the final models were calculated for each outcome with random effect terms for state.

The modeling process involved three steps. First, separate PTB and LBW models were constructed with all four WASH variables and *a priori* selected confounders (Model 1: WASH Only) to assess individual WASH-adjusted effects and confidence intervals. Second, the four social exposure variables were added to each model (Model 2: WASH-Social) to assess whether social conditions were associated with birth outcomes and whether they altered the associations between WASH exposures and outcomes. Third, interaction terms for social and WASH variables were individually added and assessed for evidence of statistically significant (p<0.05) effect modification. If interaction terms were not significant, they were removed from the fully adjusted Model 2 WASH-Social Model. However, the statistical significance of interactions product terms in nonlinear models can be misleading [47]. A simple summary measure of the interaction effect can be difficult to interpret in nonlinear models because it may include different signs for different observations. Therefore, we also examined the stratified effects and Bonferroni-corrected confidence intervals of interaction terms where there was a trend towards significance (p<0.1) or theoretical potential for effect modification. However, we limit conclusions of these subgroup results to changes in effect sizes and highlight situations where effects may be false negatives or inaccurate due to lack of power for small group size.

## Results

**Fig 1:**
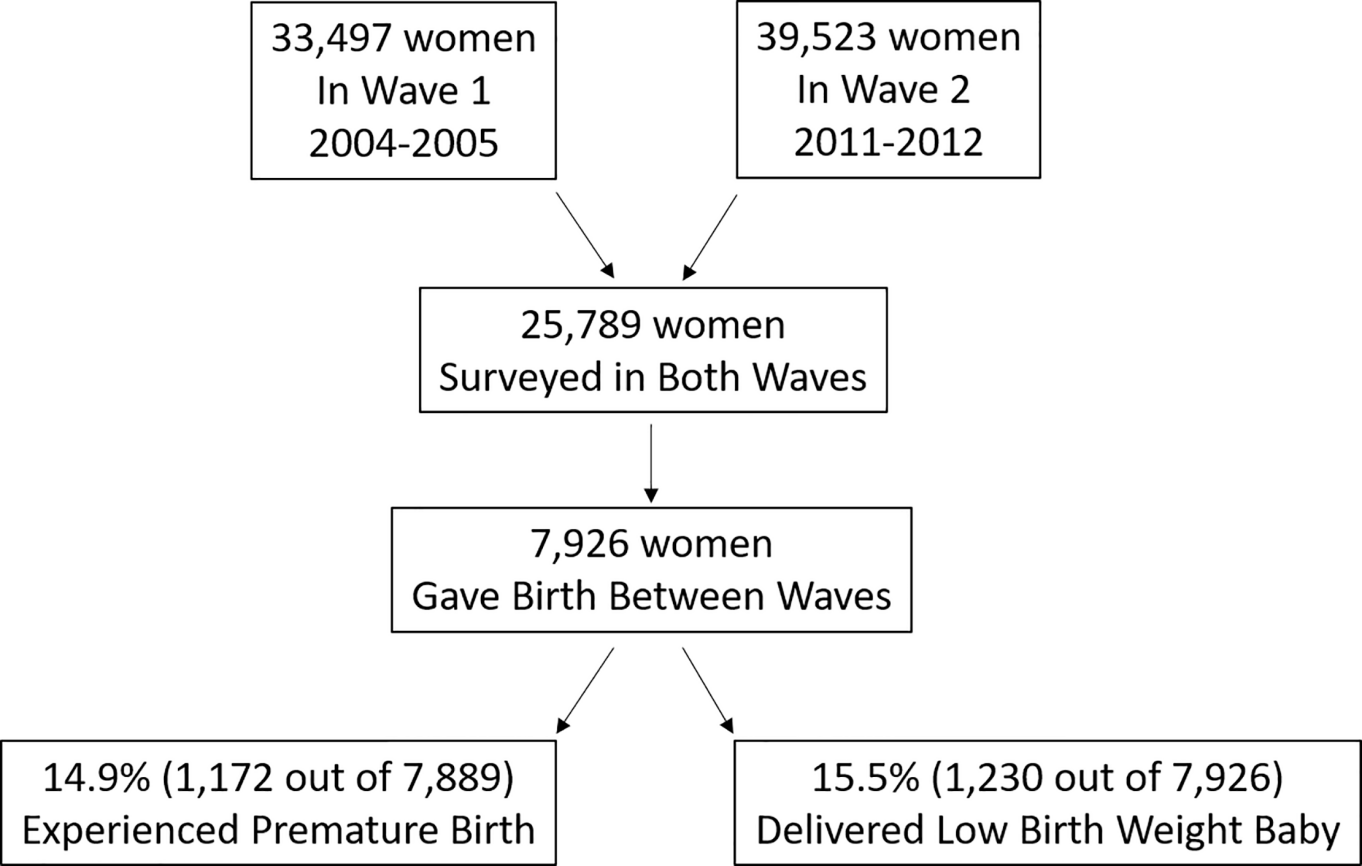
Flow diagram showing selection process for women included in this study.

Of 25,789 women who participated in both waves of the IHDS, 7,926 (30.73%) women delivered an infant between 2004/2005 and 2011/2012 and thus were eligible for this study (Fig 1). PTB and LBW infants occurred in 14.86% (1,172 out of 7,889) and 15.52% (1,230 out of 7,926) women, respectively. Both outcomes occurred jointly in 312 (3.95%) of women, which suggests that PTB and LBW are distinct outcomes. Participating women lived in 2,098 villages and urban blocks from across India (range 1–23 per village), in 360 Districts (range 1–87 per district), in 22 States (22–1,097 per state). This represents 56.3% of India’s 640 Districts and 61.1% of 36 States in 2011.

The sample population represented a diverse socio-economic background (Table 1). Women were predominantly Hindu (79.22%) and rural (71.70%). Almost half (49.80%) lacked a primary school education. At least 87.38% of women attended one or more antenatal care visits, and 78.71% of women reported taking iron supplements at some point while pregnant, with 46.10% taking supplements for three or more months. Average age was 28, but women as young as 15 and as old as 60 were included in the analytic sample. The average number of children prior to the last pregnancy was two, with a range of zero to 15.

**Table 1:** Descriptive statistics on variables used in the study.

Variable	Strata	% or Median (range)	Counts	Missing
**OUTCOME VARIABLES**				
Premature birth	Premature birth Normal birth	14.86	7,889	40
Perceived low birth weight	Small or very small Normal or above average	15.52	7,929	0
**ENVIRONMENTAL VARIABLES**				
Type drinking water source	Piped water source Hand pump or tube well Hand dug well River, canal, pond, stream Other (rainwater, truck)	38.44 44.94 14.02 1.06 1.54	3,047 3,562 1,111 84 122	0
Walking time to drinking water source	0 minutes (water in home) > 0 and ≤ 15 minutes > 15 minutes	50.29 42.68 7.03	3,980 3,378 556	12
Time spent fetching water	< 2 hours ≥ 2 hours	92.34 7.66	7,208 600	120
Sanitation access	Private household toilet Shared toilet in building/compound Shared toilet outside building/compound Open defecation	38.28 2.18 6.65 52.89	3,021 127 525 4,174	34
**SOCIAL VARIABLES**				
Harassment of women and girls	Women rarely/never harassed in community Women sometimes/often harassed in community	87.16 12.84	6,860 1,011	55
Local crime	Did not experience crime Experienced attack/threat, break-in, or theft within last 12 months	93.50 6.50	7,398 514	14
Collective efficacy	Solve problems as community Solve problems within family	58.52 41.48	4,621 3,276	29
Social cohesion	No conflict Some/lot of conflict in community	49.39 50.61	3,895 3,992	39
**POTENTIAL CONFOUNDERS**				
Geography	Rural Urban Urban Slum	71.72 26.32 1.96	5,660 2,077 155	34
Household wealth index rank	Wealthiest Upper-middle Lower-middle Poorest	24.16 28.61 23.40 23.82	1,915 2,268 1,855 1,888	0
Female education index	Completed secondary Completed primary Less than primary or none	19.57 30.63 49.80	1,541 2,412 3,921	52
Religion	Hindu Other	79.22 20.78	6,279 1,647	0
Mother’s age	Continuous	28 (15, 60)	7,926	0
Age at menarche	Continuous	14 (9, 25)	7,926	0
Parity	Continuous	2 (1, 15)	7,926	0
History of stillbirth	Yes No	5.68 94.32	7,926	0
Antenatal checkups	Any None	87.38 12.62	6,912 998	16
Iron supplements during pregnancy	>3 Months <3 Months No use	46.10 32.61 21.29	3,452 2,442 1,594	438

Most households (83.38%) had access to a piped tap, borehole, or tube well water source, with only 1.06% reporting use of some form of surface water (Table 1). Seven percent had to walk more than fifteen minutes each way to fetch drinking water and 7.66% spent more than two hours per day fetching water. A third (38.28%) of women had access to an improved household flush or Ventilated Improved Pit (VIP) latrine, 2.18% shared a latrine in their compound or building, 6.65% shared a communal or public latrine outside their building, and 52.89% relied on open defecation. Among social exposures that could cause physical harm or stress for women, 6.50% reported having experienced theft, breaking and entering to the household, or a violent attack or threatening behavior in the household. Harassment of girls and women in the community was reported by 12.84% of women. More than half of women (50.61%) reported lots of conflict within the community. Less than half of women (41.48%) reported that when there are community problems, such as with the water supply, families solved their problems by themselves, rather than bonding together with their neighbors to solve the problem.

Approximately 11.1% of the variability in PTB prevalence (ICC = 0.111) was accounted for by state-level factors, leaving 88.9% of the variability to be accounted for by other factors. In the model assessing the effects of WASH conditions on PTB, type of water source, time spent walking one way to a drinking water source, spending more than two hours per day fetching water, and type of sanitation access were not significantly associated with premature delivery, after adjusting for potential confounders (Table 2, Model 1). After adjusting for confounders and social conditions, women who shared a toilet or latrine within their building or compound were more likely to report their last child was premature than women with private facilities, while women using a public or communal facility were less likely to report premature delivery (Table 2, Model 2). The odds of premature delivery were similar for women relying on private facilities versus open defecation. Women who reported problems with local crime in the community, or harassment of women and girls were also more likely to report premature delivery compared to women who reported that crime or harassment were not community issues. Social cohesion and collective efficacy were not associated with PTB.

**Table 2:** Adjusted odds ratios and 95% confidence intervals for associations between water, sanitation, and social conditions and PTB outcomes in 7,105 women between 2004/5 and 2011/2012 waves of the IHDS.

Exposure	Category (reference)	Model 1: WASH Only OR (95% CI)	Model 2: WASH—Social OR (95% CI)	Model 3: Adjusted Interactions OR (Bonferroni 95% CI)
**Environmental Variables**				
**Drinking Water Source**	Piped Water	**Ref.**	Ref.	
	Ground water (hand pump or tube well)	1.06 (0.87, 1.30)	1.06 (0.87, 1.29)	1.06 (0.87, 1.29)
	Hand dug well	0.93 (0.71, 1.21)	0.93 (0.71, 1.22)	0.94 (0.72, 1.23)
	Stream, pond, river, canal	0.44 (0.17, 1.13)	0.45 (0.18, 1.15)	0.45 (0.18, 1.16)
	Other (tanker truck, rainwater, other)	0.79 (0.40, 1.57)	0.83 (0.42, 1.65)	0.83 (0.42, 1.66)
**Time spent fetching water**	≥ 2 hours per day (<2 hours per day)	0.89 (0.67, 1.18)	0.88 (0.66, 1.18)	0.87 (0.65, 1.16)
**Walking time to water**	Water in home (0 minutes)	Ref.	Ref.	Ref.
	1–15 minutes one way to water	0.98 (0.83, 1.17)	1.00 (0.85, 1.19)	1.01 (0.85, 1.20)
	>15 minutes one way to water	1.08 (0.78, 1.50)	1.09 (0.78, 1.51)	1.09 (0.79, 1.52)
**Sanitation access**	Private household latrine	Ref.	Ref.	Ref.
	Shared latrine, within building or compound	1.50 (0.98, 2.31)	**1.55 (1.01, 2.38)**	Family: 0.91 (0.39, 2.14) **Community: 2.52 (1.17, 5.45)**
	Shared latrine, outside building or compound or Public latrine	0.68 (0.46, 1.02)	0.67 (0.45, 1.01)	Family: 0.61 (0.31, 1.19) Community: 0.71 (0.31, 1.63)
	Open defecation	1.05 (0.86, 1.28)	1.03 (0.84, 1.26)	Family: 0.86 (0.60, 1.23) Community: 1.16 (0.84, 1.59)
**Social Variables**				
**Harassment of women and girls**	Yes (No)		**1.33 (1.09, 1.62)**	**1.33 (1.09, 1.62)**
**Local crime**	Yes (No)		**1.30 (1.00, 1.68)**	1.29 (0.99, 1.67)
**Collective efficacy**	Act with Family (Act with Community)		0.97 (0.84, 1.12)	Effect Modifier
**Social cohesion**	Conflict (No Conflict)		0.91 (0.79, 1.06)	0.91 (0.79, 1.06)
**-2LogL (Generalized Chi-Square/DF)**		36,716.32 (0.99)	35,399.20 (1.00)	36,358.10 (0.98)

• Premature birth model with spatial random effects only -2Log L = 39,736.04. Adjusted for geography, household wealth index rank, maternal education, religion, maternal age, maternal age at menarche, parity, history of stillbirth, health care utilization for antenatal care, and use of iron supplements during pregnancy. Model 3 adjusted for interaction term between sanitation access and collective efficacy. Associations for confounders reported in S2 Table.

Collective efficacy modified the association between sanitation access and PTB (p = 0.047, S3 Table). Subgroup analysis revealed that sharing a within-building or compound latrine was not significantly associated with PTB for women who solved problems with their families, but was strongly associated for women who solved water problems with their community (Table 2, Model 3). Weak interaction (p = 0.08) was observed between local crime and one-way time to fetch water. Stratified analysis revealed that PTB was not associated with walking 1 to 15 minutes (adjOR = 0.97; Bonferonni-corrected 95% CI = 0.78, 1.22) or >15 minutes (adjOR = 1.00; Bonferonni-corrected 95% CI = 0.64, 1.55) one-way to fetch drinking water in communities where crime was uncommon. However, the association between PTB and walking 1 to 15 minutes (adjOR = 1.38; Bonferonni-corrected 95% CI = 0.74, 2.59) or >15 minutes (adjOR = 3.09; Bonferonni-corrected 95% CI = 0.88, 10.86) one-way to fetch drinking water was much larger for women in communities where crime was common. Another interesting subgroup analysis found that harassment of women and girls and sanitation access was significantly associated with PTB among women who practiced open defecation (adjOR = 1.49; 95% CI = 1.09, 2.05), but not for women using private household latrines (adjOR = 1.12; 95% CI = 0.73, 1.73), or within-building/compound shared latrines (adjOR = 1.49; 95% CI = 0.33, 6.71), or shared public/communal latrines (adjOR = 0.92; 95% CI = 0.28, 3.00).

Approximately 5.6% of the variability in low infant birth weight prevalence (ICC = 0.056) was accounted for by state-level factors, leaving 94.4% of the variability to be accounted for by other factors. Spending more than two hours per day fetching water was associated with increased odds of delivering a LBW infant, after adjusting for other WASH conditions as well as potential confounders (Table 3, Model 1). Type of primary water source, time spent walking to the drinking water source, and type of sanitation access were not significantly associated with LBW. After adjusting for social conditions and other potential confounders, the direction and size of effects and confidence intervals for WASH conditions and LBW remained similar, although open defecation was more strongly associated with higher odds of LBW (Table 3, Model 2). Among the social variables, harassment of women and girls was significantly associated with LBW, while general local crime, collective efficacy, and social cohesion were not associated.

**Table 3:** Adjusted odds ratios and 95% confidence intervals for associations between water, sanitation, and social conditions and low birth weight outcomes in 7,177 women between 2004/5 and 2011/2012 waves of the IHDS.

Exposure	Category (reference)	WASH Only OR (95% CI)	Full Model OR (95% CI)
**Environmental Variables**			
**Drinking Water Source**	Piped Water	Ref.	Ref.
	Ground water (hand pump or tube well)	1.18 (0.98, 1.42)	1.17 (0.97, 1.41)
	Hand dug well	0.85 (0.66, 1.09)	0.82 (0.64, 1.05)
	Stream, pond, river, canal	0.64 (0.32, 1.31)	0.63 (0.31, 1.28)
	Other (tanker truck, rainwater, other)	1.24 (0.72, 2.14)	1.28 (0.74, 2.21)
**Time spent fetching water**	≥ 2 hours per day (<2 hours per day)	**1.31 (1.03, 1.67)**	**1.33 (1.05, 1.70)**
**Walking time to water**	(0 minutes to water [water in home])	Ref.	Ref.
	0–15 minutes to water	0.95 (0.80, 1.12)	0.95 (0.81, 1.12)
	>15 minutes to water	0.96 (0.72, 1.28)	0.97 (0.72, 1.29)
**Sanitation access**	Private household latrine	Ref.	Ref.
	Shared latrine in building/compound	0.84 (0.50, 1.39)	0.86 (0.52, 1.44)
	Shared latrine, outside building/compound or Public latrine	1.05 (0.77, 1.44)	1.02 (0.74, 1.40)
	Open defecation	1.23 (0.72, 1.49)	**1.22 (1.00, 1.48)**
**Social Variables**			
**Harassment of women and girls**	Yes (No)		**1.26 (1.03, 1.54)**
**Local crime**	Yes (No)		1.13 (0.87, 1.48)
**Collective efficacy**	Act with Family (Act with Community)		0.96 (0.84, 1.11)
**Social cohesion**	Conflict (No Conflict)		0.93 (0.81, 1.07)
**-2 Log L (Generalized Chi-Square/DF)**		35,753.11 (1.00)	35,396.46 (1.00)

• Low birth weight model with spatial random effects only -2Log L = 38,860.76. Adjusted for geography, household wealth index rank, maternal education, religion, maternal age, maternal age at menarche, parity, history of stillbirth, health care utilization for antenatal care, and use of iron supplements during pregnancy. Associations for confounders reported in S4 Table.

No statistically significant effect modification was detected for WASH and social conditions on LBW, although weak interaction (p = 0.092) was detected between spending > 2 hours per day fetching water and collective efficacy (S5 Table). Subgroup assessment of the association between hours per day fetching water and LBW suggested that the association between increased water fetching time (> 2 hours/day) and LBW was higher in households that solved water problems as a community (adjOR = 1.54; 95% CI = 1.10, 2.14) than households that solved problems as a family (adjOR = 1.02; 95% CI = 0.64, 1.62).

## Discussion

Preterm birth and low infant birth weight are among the leading causes of neonatal and early childhood morbidity and mortality in LMICs, like India, and prevalence rates in Africa and Southeast Asia are rising [1–3]. The rising rates of PTB and LBW in LMICs underscore the importance of identifying targeted interventions that prevent these birth outcomes and subsequent child morbidity and mortality. However, the wide range of biological, environmental, and social risk factors complicates identifying preventive interventions for PTB and LBW. This study contributes to the limited evidence related to environmental causes of PTB and LBW by demonstrating that lack of household WASH infrastructure and social factors, like crime and harassment of women and girls, are risk factors for adverse birth outcomes in women in LMICs. In particular, this study uses a large, nationally representative cohort of Indian women to demonstrate the importance of the lack of access to nearby water sources, lack of access to a toilet, crime, and harassment as risk factors for PTB and LBW. Additionally, the findings suggest that gender norms that sanction harassment of women and girls and place the burden of household water fetching on women are key determinants of vulnerability to PTB and LBW among Indian women.

The lack of water and sanitation in the household forces women to navigate challenging, and even personally threatening social and environmental public conditions to fetch water and to find a safe, private place to defecate, bathe, or manage menstruation, leading to psychosocial stress [21, 22, 43]. Our findings reinforce evidence from two prior studies that open defecation, shared sanitation, and infrequent latrine use increase the risk of adverse birth outcomes in Indian and Nigerian women, but provides new potential explanations for the mechanism of these associations [17, 18]. Our results suggest a role for both physical and psychosocial stress as mechanisms for adverse birth outcomes. We found that increased time daily spent fetching household water increased women’s risk of a LBW outcome. Spending over two hours per day carrying water from sources outside of the home may increase the risk for injury, physical exhaustion, and maternal and fetal nutrient restriction, or psychosocial stress, depression or anxiety [24, 30]. Work itself is not necessarily dangerous for pregnant women, although physical labor, standing for many hours per day, working an unusually high number of hours per day, occupational fatigue, and having many domestic dependents with no domestic support increase PTB and LBW risks [48–50]. Regardless, this finding suggests reducing the water-fetching burden could reduce LBW prevalence in Indian pregnant women, a condition resolved by providing a reliable household water source.

Open defecation and using a shared latrine within a woman’s building or compound were also associated with higher odds of LBW and PTB, respectively, compared to having a private household toilet. Lack of access to a private toilet forces women to cope with urination, defecation, and menstruation needs in public areas, which increases the amount of time they could experience harassment [21, 30]. The time and energy spent seeking a safe, private place to open defecate could increase the risk of LBW through physical stress, injury, or fatigue. Due to the proximity of compound-shared latrines, this relationship with PTB is not likely due to physical stress, unless women relying on shared latrines use them infrequently (only 2% of our sample population reported using a compound-shared latrine) and experience similar exposures as women who rely on open defecation [51, 52]. The infrequent use of latrines in India is due, in part, to the stigma associated with handling feces [53]. Therefore, there may be confounding socioeconomic or cultural factors associated with the use of a shared latrine. For example, in urban Nigeria where sharing a sanitation facility was also found to be a risk factor for PTB, shared sanitation was used as an indicator of poor housing conditions [17]. Alternatively, sharing a within-compound latrine requires cooperation among families in maintaining those latrines, and this association might reflect stress from negotiating latrine management relationships or increased workload for women in maintaining shared latrines. Oddly, the relationship between sharing public or community latrines and PTB trended in the opposite direction. An extensive array of factors (e.g. urban/rural geography, wealth, crime) were examined and ruled out as confounders or modifiers of this effect. Our theory is that while reliance upon public or community latrines does cause stress to women [21, 22], perhaps it significantly alleviates the stressors that trigger PTB. For example, public latrine users may bear less responsibility to clean and maintain infrastructure or experience less social conflict over their upkeep [43]. Further research is needed to understand the underlying reason for the association between shared latrine use and PTB in the Indian context.

Our findings demonstrate that poor community-level social conditions are independently associated with both PTB and LBW outcomes, regardless of household WASH conditions, and modify the impact of WASH access on those outcomes. Women who reported that gender-based harassment was common in their community had higher odds of PTB and LBW, and those who reported issues with local crime (i.e., an attack/threat, break-in, or theft) were trending towards statistically higher odds of PTB. This expands the evidence in LMICs on the association between maternal stress and birth outcomes beyond intimate partner violence [54]. Although harassment and crime (often perpetrated by strangers) are different from violence committed by an intimate partner, both forms of abuse lead to increased levels of psychosocial stress, which is a known risk factor for PTB and LBW [33, 35]. More research is needed to better understand the impact of harassment on maternal stress and adverse birth outcomes, especially in areas where harassment towards women is common and often considered socially acceptable [55, 56].

To elucidate the mechanisms through which sources of physical stress and psychosocial stress affect birth outcomes, we examined the interaction between WASH access and a variety of social factors. We found trends suggesting that fetching water outside the household and open defecation were associated with adverse birth outcomes only when community-level crime and gender-based harassment were common, respectively. The effect size for time to a primary drinking water source on PTB grew with increases in one-way walk time compared to women with a household source among women who reported crime, but had a null effect on women who did not report crime in their community. The effect size for harassment on PTB was 33% larger for women sharing a building/compound latrine or practicing open defecation compared to women with private sanitation, suggesting lack of sanitation increases women’s vulnerability to harassment. These relationships were not statistically implicated, due, in part, to small sample sizes for some WASH categories caused by subgroup analysis. Effect sizes for harassment and LBW did not vary across sanitation categories, suggesting that physical stress or open defecation-related risks (other than harassment) contribute to fetal growth restriction. Although coping with uneven terrain, monsoon conditions, and wildlife may further contribute to the set of stressors experienced by women who open defecate, ultimately harassment plays a dominant role in how sanitation affects birth outcomes in this study.

We found effect modification by collective efficacy of sanitation for PTB as well as trends that suggested that collective efficacy modified the effect of water fetching on LBW. Specifically, using a within-compound shared latrine was a risk factor for PTB among women who relied on their community to resolve water problems (an indicator of collective efficacy), but not among women who solved problems within their own family. This finding was unexpected, but has two possible explanations. First, it could indicate unmeasured benefits from support by one’s family in managing a shared latrine. Family-level support could reduce the likelihood of depressive symptoms in women, and therefore likelihood of PTB [57, 58], or it could represent better marital relationships and better access to care [59] and a lower likelihood of domestic violence [60]. Second, women who relied on their community to resolve water problems may be an indicator of women living in communities with more water problems instead of an indicator of collective efficacy. In a previous study on the measurement of social capital, it was found that questions about collective action were correlated with negative community attributes due to the way the question was asked [61]. This is a limitation of the study since we do not have the capacity to change the way the question was asked. Similarly, the association between water fetching and LBW occurred only among women who solved problems with their community rather than their own family, potentially reflecting unmeasured benefits of family support for water access.

Additional limitations of our study, beyond power to conduct subgroup analysis, include the use of self-reported birth outcomes, which may be subject to recall or courtesy bias, and limited information on women’s social experiences related to water and sanitation. Despite the data limitations, this is the only data set would allow us to examine environmental and social aspects of WASH access and birth outcomes. Another limitation was the lack of information on potential maternal confounders, such as maternal BMI and reproductive tract infections. Alternative mechanisms associated with unsafe WASH access that could explain our findings include higher risks of malarial, helminthic, or diarrheal infections due to increased contact with public soils and surface waters, or contact with environmental chemical pollutants. Additionally, women with limited WASH access may practice unsafe hygiene behaviors that increase their risk for intrauterine infection by foreign microbes (e.g. *Gardnerella vaginalis*, *Candida spp*. or *Mycoplasma spp*.) that can stimulate myometrium contractions and/or the rupture of chorioamniotic membranes [23, 62]. Information on these health conditions were not available in this dataset. Gender-based harassment may have been underreported [63], but we believe this would be non-differential misclassification and would not affect our findings. Caste may have been an important confounder, but we could not adjust for caste without excluding a large number of participants who may be from socially-marginalized groups. Last, this observational study cannot confirm causality and is vulnerable to bias and residual confounding.

Maternal psychosocial stress, anxiety, and depression—due to a combination of environmental and social factors—are preventable risk factors for PTB and LBW. In HICs, stress has been linked to domestic conditions like unemployment, poverty, and domestic violence, as well as community-level social conflict and environmental resource deprivation [28, 29]. There is very little research on stress risk factors and maternal reproductive health outcomes in LMICs, although physical and psychosocial stress is an omnipresent part of daily life where extreme levels of poverty require households to cooperate in sharing infrastructure, like water sources and toilets. Women’s ability to meet sanitation needs is limited by both domestic labor constraints and by capacity to find places outside the home that are safe and private, and gaining access to a latrine may not eliminate stress, so much as exchange one set of stressors for others [43]. Based on our findings, there is a need to more closely examine the pathways through which lack of WASH access and harassment interact to impact maternal stress and, subsequently, birth outcomes. Additionally, interventions that build social capital and change social norms related to gender-based causes of maternal stress in the context of WASH behaviors need to be evaluated, especially in locations with high levels of gender inequity.

## Supporting information

S1 Table Variables used in this analysis. (DOCX)

S2 Table Adjusted odds ratios and 95% confidence intervals for confounder variables included in Table 2 analysis of associations between water, sanitation, and social conditions and preterm birth outcomes in 7,105 women between 2004/5 and 2011/2012 waves of the IHDS. (DOCX)

S3 Table Interaction term effects tested in the Model 2 analysis of water, sanitation, and social conditions and premature birth outcomes in 7,105 women between 2004/5 and 2011/2012 waves of the IHDS. Did not converge (NC).
(DOCX)

S4 Table Adjusted odds ratios and 95% confidence intervals for confounder variables included in Table 2 analysis of associations between water, sanitation, and social conditions and low birth weight outcomes in 7,177 women between 2004/5 and 2011/2012 waves of the IHDS. (DOCX)

S5 Table Interaction term effects tested in the Model 2 analysis of water, sanitation, and social conditions and low infant birth outcomes in 7,177 women between 2004/5 and 2011/2012 waves of the IHDS. Did not converge (NC).
(DOCX)
