# Supplementary material for: Impact of social capital, harassment of women and girls, and water and sanitation access on premature birth and low infant birth weight in India
Source: PLoS One. 2018 Oct 8;13(10):e0205345. doi: 10.1371/journal.pone.0205345 (PMC6175511; doi:10.1371/journal.pone.0205345)
Supplement: S1 Table — (DOCX) [file pone.0205345.s001.docx]

S1 Table. Variables used in this analysis.

| Variable | Type | Variable Name(s) | Classification Definition |
| --- | --- | --- | --- |
| Premature birth | Outcome | premature_birth | premature birth  normal birth |
| Perceived low birth weight | Outcome | bw_w2 | Small or very small birth weight  Normal or above average birth weight |
| Improved drinking water source | Environmental Exposure | Imp_techu | 1 = river, pond, truck, bottled, other  0 = piped, tube well, hand pump, covered well, rainwater |
| Walking time to water | Environmental Exposure | time_to_water | > 15 minutes  > 0 and ≤ 15 minutes  0 minutes (water in home) |
| Time spent fetching water | Environmental Exposure | time_water_carry | ≥ 2 hours per day  <2 hours per day |
| Sanitation access | Environmental Exposure | Improved_san_full | 0 = Traditional latrine, VIP latrine, flush toilet access  1 = Shared latrine within building/compound  2 = Shared latrine outside building/compound  or Public latrine  3 = Open defecation |
| Harassment of women and girls within community | Social Exposure | Harassment_girls | 1 = Women are harassed sometimes/often  0 = rarely/never |
| Local crime | Social Exposure | Local_crime | 1 = Within last 12 months woman experienced an attack/threat, break-in, or theft.  0 = Woman did not experience one of those events |
| Collective efficacy | Social Exposure | Collective Efficacy | 1 = Solve problems per family  0 = Band together to solve problems as community |
| Social cohesion | Social Exposure | Social_cohesion | 1 = There is a lot of conflict in the community  0 = Everyone gets along |
| Geography | Confounder | ID9 | 1 = urban slum  2 = urban  3 = rural |
| Wealth index rank | Confounder | r_hhassets | 0 to 3 in low to high wealth indices |
| Female education index | Confounder | HHED_FEMALE | . = invalid skip/blank  2=None  1 =1-9^th^ standard  0 = 10^th^ standard and above |
| Religion | Confounder | ReligSTORY | 0 = Hindu  1 = Other |
| Mother’s age | Confounder | Mother_age | Age of mother |
| Parity | Confounder | Parity | Number total births during woman’s life, including most recent child |
| Age at Menarche | Confounder | M2HC | Age at onset of menstruation |
| History of stillbirth | Confounder | Stillbirth | Any history of stillbirth versus no history of stillbirth |
| Antenatal check ups | Confounder | Antenatalcheck | no checkups  at least 1 checkup |
| Iron Supplements During Pregnancy | Confounder | Iron_tablet | >3 Months  <3 Months  No use |
| Vent in cooking area | Confounder | vented_cooking | Vented indoor cooking area  Unvented indoor cooking area  Cooks outside |
