# Supplementary material for: Impact of social capital, harassment of women and girls, and water and sanitation access on premature birth and low infant birth weight in India
Source: PLoS One. 2018 Oct 8;13(10):e0205345. doi: 10.1371/journal.pone.0205345 (PMC6175511; doi:10.1371/journal.pone.0205345)
Supplement: S2 Table — (DOCX) [file pone.0205345.s002.docx]

S2 Table. Adjusted odds ratios and 95% confidence intervals for confounder variables included in Table 2 analysis of associations between water, sanitation, and social conditions and preterm birth outcomes in 7,105 women between 2004/5 and 2011/2012 waves of the IHDS.

| **Exposure** | **Category (reference)** | **Model 1: WASH Only**  **OR (95% CI)** | **Model 2: WASH - Social**  **OR (95% CI)** |
| --- | --- | --- | --- |
| **Household Assets** | 0 (Poorest) | Ref. | Ref. |
|  | 1 | 0.96 (0.76, 1.20) | 0.98 (0.77, 1.23) |
|  | 2 | 1.09 (0.82, 1.44) | 1.12 (0.84, 1.49) |
|  | 3 (Wealthiest) | 1.17 (0.85, 1.59) | 1.18 (0.86, 1.61) |
| **Antenatal visit** | At least one visit vs. none | 0.93 (0.74, 1.17) | 0.94 (0.74, 1.18) |
| **Maternal education** | 10^th^ standard and above | Ref. | Ref. |
|  | 1-9^th^ standard | 1.11 (0.87, 1.40) | 1.10 (0.87, 1.39) |
|  | None | 1.35 (1.06, 1.73) | 1.35 (1.05, 1.73) |
| **Maternal Age (years)** | Increase of 1 year | 1.00 (0.98, 1.02) | 1.00 (0.84, 1.02) |
| **Iron tablets** | >3 Months | Ref. | Ref. |
|  | <3 Months | 1.61 (1.37, 1.88) | 1.58 (1.34, 1.85) |
|  | No use | 0.99 (0.81, 1.22) | 1.00 (0.82, 1.23) |
| **Religion** | Hindu vs. other | 1.20 (1.01, 1.43) | 1.21 (1.02, 1.45) |
| **Household servant** | Yes (No) | 0.97 (0.65, 1.45) | 0.95 (0.64, 1.43) |
| **Parity (number children)** | Increase of one birth | 0.92 (0.87, 0.97) | 0.92 (0.86, 0.97) |
| **Age at menarche** | Increase of 1 year | 1.04 (0.99, 1.10) | 1.05 (1.00, 1.10) |
| **Geography** | Urban slum | Ref. | Ref. |
|  | Urban | 0.98 (0.80, 1.19) | 0.98 (0.80, 1.19) |
|  | Rural | 1.61 (0.98, 2.65) | 1.64 (1.00, 2.70) |
| **Stillbirth** | Any history (None) | 1.36 (1.05, 1.78) | 1.38 (1.06, 1.80) |
