# Supplementary material for: Impact of social capital, harassment of women and girls, and water and sanitation access on premature birth and low infant birth weight in India
Source: PLoS One. 2018 Oct 8;13(10):e0205345. doi: 10.1371/journal.pone.0205345 (PMC6175511; doi:10.1371/journal.pone.0205345)
Supplement: S3 Table — Did not converge (NC). (DOCX) [file pone.0205345.s003.docx]

S3 Table. Interaction term effects tested in the Model 2 analysis of water, sanitation, and social conditions and premature birth outcomes in 7,105 women between 2004/5 and 2011/2012 waves of the IHDS.

| Interaction term | F value | P value | -2 Log L (Chi-Square/DF) |
| --- | --- | --- | --- |
| Full Model Only |  |  | 35399.20 (1.00) |
| Social cohesion * improved water | 1.58 | 0.18 | 36392.08 (0.99) |
| Social cohesion * Water fetching time | 2.12 | 0.15 | 36342.23 (0.98) |
| Social cohesion * Time to Water | 1.09 | 0.34 | 36346.50 (0.98) |
| Social cohesion * Sanitation access | 1.45 | 0.22 | 36361.96 (0.99) |
| Collective efficacy * improved water | 0.73 | 0.57 | 36344.84 (0.98) |
| Collective efficacy * Water fetching time | 1.04 | 0.31 | 36335.79 (0.98) |
| Collective efficacy * Time to Water | 1.89 | 0.15 | 36361.13 (0.99) |
| **Collective efficacy * Sanitation access** | **2.65** | **0.047** | **36358.10 (0.98)** |
| Local crime * improved water | NC | - | - |
| Local crime * Water fetching time | 1.40 | 0.24 | 36336.48 (0.98) |
| **Local crime * Time to Water** | **2.53** | **0.08** | **36352.31 (0.99)** |
| Local crime * Sanitation access | 1.42 | 0.23 | 36333.28 (0.98) |
| Harassment of women and girls * improved water | NC | - | - |
| Harassment of women and girls * Water fetching time | 1.81 | 0.18 | 36343.33 (0.98) |
| Harassment of women and girls * Time to Water | 0.48 | 0.62 | 36340.20 (0.98) |
| Harassment of women and girls * Sanitation access | 0.85 | 0.47 | 36340.44 (0.99) |

Did not converge (NC).
