# Supplementary material for: Impact of social capital, harassment of women and girls, and water and sanitation access on premature birth and low infant birth weight in India
Source: PLoS One. 2018 Oct 8;13(10):e0205345. doi: 10.1371/journal.pone.0205345 (PMC6175511; doi:10.1371/journal.pone.0205345)
Supplement: S4 Table — (DOCX) [file pone.0205345.s004.docx]

S4 Table. Adjusted odds ratios and 95% confidence intervals for confounder variables included in Table 2 analysis of associations between water, sanitation, and social conditions and low birth weight outcomes in 7,177 women between 2004/5 and 2011/2012 waves of the IHDS.

| **Exposure** | **Category (reference)** | **Model 1: WASH Only**  **OR (95% CI)** | **Model 2: WASH - Social**  **OR (95% CI)** |
| --- | --- | --- | --- |
| **Household Assets** | 0 (Poorest) | Ref. | Ref. |
|  | 1 | 1.15 (0.92, 1.44) | 1.18 (0.94, 1.48) |
|  | 2 | 1.27 (0.97, 1.66) | 1.31 (1.00, 1.72) |
|  | 3 (Wealthiest) | 1.31 (0.97, 1.77) | 1.33 (0.98, 1.80) |
| **Antenatal visit** | At least one visit vs. none | 1.01 (0.81, 1.26) | 1.00 (0.80, 1.25) |
| **Maternal education** | 10^th^ standard and above | Ref. | Ref. |
|  | 1-9^th^ standard | 0.97 (0.78, 1.21) | 0.95 (0.76, 1.19) |
|  | None | 0.97 (0.76, 1.22) | 0.96 (0.76, 1.21) |
| **Maternal Age (years)** | Increase of 1 year | 0.97 (0.95, 0.98) | 0.97 (0.95, 0.98) |
| **Iron tablets** | >3 Months | Ref. | Ref. |
|  | <3 Months | 1.33 (1.14, 1.55) | 1.31 (1.12, 1.53) |
|  | No use | 1.29 (1.06, 1.56) | 1.29 (1.06, 1.57) |
| **Religion** | Hindu vs. other | 0.97 (0.81, 1.16) | 0.98 (0.82, 1.18) |
| **Household servant** | Yes (No) | 0.99 (0.66, 1.48) | 1.01 (0.67, 1.53) |
| **Parity (number children)** | Increase of one birth | 1.07 (1.01, 1.13) | 1.07 (1.01, 1.13) |
| **Age at menarche** | Increase of 1 year | 1.00 (0.95, 1.04) | 1.00 (0.95, 1.05) |
| **Geography** | Urban slum | Ref. | Ref. |
|  | Urban | 1.08 (0.89, 1.30) | 1.06 (0.87, 1.29) |
|  | Rural | 1.49 (0.93, 2.39) | 1.43 (0.88, 2.31) |
| **Stillbirth** | Any history (None) | 0.99 (0.75, 1.31) | 0.98 (0.74, 1.30) |
