# Supplementary material for: Impact of social capital, harassment of women and girls, and water and sanitation access on premature birth and low infant birth weight in India
Source: PLoS One. 2018 Oct 8;13(10):e0205345. doi: 10.1371/journal.pone.0205345 (PMC6175511; doi:10.1371/journal.pone.0205345)
Supplement: S5 Table — Did not converge (NC). (DOCX) [file pone.0205345.s005.docx]

S5 Table. Interaction term effects tested in the Model 2 analysis of water, sanitation, and social conditions and low infant birth outcomes in 7,177 women between 2004/5 and 2011/2012 waves of the IHDS.

| Interaction term | F value | P value |
| --- | --- | --- |
| Social cohesion * improved water | 0.71 | 0.58 |
| Social cohesion * Water fetching time | 0.02 | 0.89 |
| Social cohesion * Time to Water | 0.15 | 0.86 |
| Social cohesion * Sanitation access | 1.52 | 0.21 |
| Collective efficacy * improved water | 1.02 | 0.40 |
| **Collective efficacy * Water fetching time** | **2.84** | **0.09** |
| Collective efficacy * Time to Water | 0.35 | 0.71 |
| Collective efficacy * Sanitation access | 0.20 | 0.90 |
| Local crime * improved water | NC | NC |
| Local crime * Water fetching time | 0.73 | 0.39 |
| Local crime * Time to Water | 0.87 | 0.42 |
| Local crime * Sanitation access | 0.26 | 0.85 |
| Harassment of women and girls * improved water | NC | NC |
| Harassment of women and girls * Water fetching time | 0.06 | 0.81 |
| Harassment of women and girls * Time to Water | 0.42 | 0.66 |
| Harassment of women and girls * Sanitation access | 1.40 | 0.24 |

Did not converge (NC).
